# Preoperative transcranial magnetic stimulation for picture naming is reliable in mapping segments of the arcuate fasciculus

**DOI:** 10.1093/braincomms/fcaa158

**Published:** 2020-09-29

**Authors:** Davide Giampiccolo, Henrietta Howells, Ina Bährend, Heike Schneider, Giovanni Raffa, Tizian Rosenstock, Francesco Vergani, Peter Vajkoczy, Thomas Picht

**Affiliations:** Department of Neurosurgery, Verona University Hospital, University of Verona, Verona, Italy; Department of Neurosurgery, Charité University Hospital, Berlin, Germany; MoCa Laboratory, University of Milan, Milan, Italy; Department of Neurosurgery, Charité University Hospital, Berlin, Germany; Department of Neurosurgery, Charité University Hospital, Berlin, Germany; Department of Neurosurgery, Messina University Hospital, Italy; Department of Neurosurgery, Charité University Hospital, Berlin, Germany; Department of Neurosurgery, King’s College Hospital NHS Foundation Trust, London, UK; Department of Neurosurgery, Charité University Hospital, Berlin, Germany; Department of Neurosurgery, Charité University Hospital, Berlin, Germany

**Keywords:** language, TMS, tumour, tractography, arcuate

## Abstract

In preoperative planning for neurosurgery, both anatomical (diffusion imaging tractography) and functional tools (MR-navigated transcranial magnetic stimulation) are increasingly used to identify and preserve eloquent language structures specific to individuals. Using these tools in healthy adults shows that speech production errors occur mainly in perisylvian cortical sites that correspond to subject-specific terminations of the major language pathway, the arcuate fasciculus. It is not clear whether this correspondence remains in oncological patients with altered tissue. We studied a heterogeneous cohort of 30 patients (fourteen male, mean age 44), undergoing a first or second surgery for a left hemisphere brain tumour in a language-eloquent region, to test whether speech production errors induced by preoperative transcranial magnetic stimulation had consistent anatomical correspondence to the arcuate fasciculus. We used navigated repetitive transcranial magnetic stimulation during picture naming and recorded different perisylvian sites where transient interference to speech production occurred. Spherical deconvolution diffusion imaging tractography was performed to map the direct fronto-temporal and indirect (fronto-parietal and parieto-temporal) segments of the arcuate fasciculus in each patient. Speech production errors were reported in all patients when stimulating the frontal lobe, and in over 90% of patients in the parietal lobe. Errors were less frequent in the temporal lobe (54%). In all patients, at least one error site corresponded to a termination of the arcuate fasciculus, particularly in the frontal and parietal lobes, despite distorted anatomy due to a lesion and/or previous resection. Our results indicate that there is strong correspondence between terminations of the arcuate fasciculus and speech errors. This indicates that white matter anatomy may be a robust marker for identifying functionally eloquent cortex, particularly in the frontal and parietal lobe. This knowledge may improve targets for preoperative mapping of language in the neurosurgical setting.

## Teaser

Navigated transcranial magnetic stimulation for mapping language areas is increasingly used in presurgical planning for personalized brain tumour treatment. Giampiccolo *et al.* use tractography to show arcuate fasciculus terminations match speech-eloquent cortex, even in patients with distorted anatomy.

## Graphical Abstract

**Graphical Abstract:**
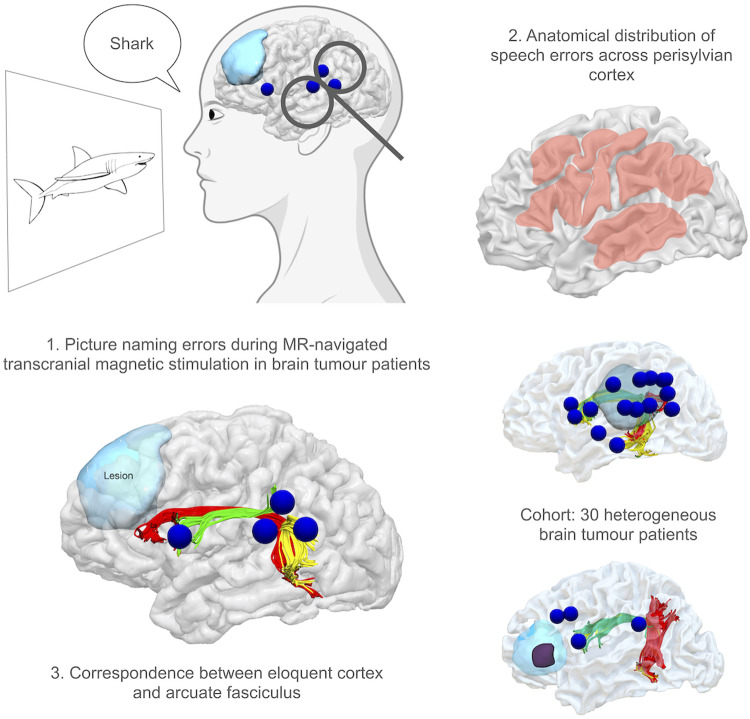

## Introduction

Preservation of function is an essential goal in neuro-oncology. Permanent language deficits are a major concern when planning the surgical removal of tumours in eloquent areas, as deficits have a profound effect on patients’ quality of life: affecting ability to return to work, social ability and mood (Ferro and Madureira, 1997). A key challenge is the unpredictability of the location of essential language sites, as evaluated from anatomical landmarks. To overcome this, extensive presurgical examinations and intraoperative mapping using direct electrical stimulation (DES) in the awake neurosurgical setting are advocated to test eloquent areas (Bello *et al.*, 2007). Operating on awake patients requires a complex setting, necessitating good patient compliance, a trained neuropsychologist and tailored anaesthesia protocols. Therefore, reliable preoperative techniques to identify language-eloquent regions would be of great value to the neurosurgical community.

Functional magnetic resonance imaging is commonly used in the preoperative phase, but its reliability has been questioned as the effect of damaged tissue on brain haemodynamics is unknown (Giussani *et al.*, 2010). Transcranial magnetic stimulation (TMS) offers a causal but non-invasive means by which to test cortical regions involved in a given function, by applying a magnetic field to the scalp, electrically inducing a physiological response in brain tissue (Rossi *et al.*, 2012). Repetitive TMS produces a focal transient virtual lesion that disrupts brain function to cause behavioural changes, that is somewhat comparable to DES (Lioumis *et al.*, 2012). In healthy subjects, TMS using different picture naming paradigms has significantly extended the understanding of the neurobiological underpinnings of different components of speech production (Devlin and Watkins, 2007). Despite this, its reliability for preoperative planning has been heavily debated due to both behavioural and anatomical interindividual variability (Michelucci *et al.*, 1994). One limitation has been that although this disruption is focal, it has traditionally been applied using cranial landmarks, thus it has been unable to evaluate differences in cortical architecture. Recent technological upgrades enable real-time tracking of the induced electrical field alongside the patient’s structural MRI, termed e-field navigated transcranial magnetic stimulation (nTMS) (Ruohonen and Karhu, 2010). This approach provides more anatomically specific stimulation targets for preoperative neurosurgical mapping of language-eloquent regions, and is a rapidly growing field within personalized medicine.

Using repetitive nTMS with an object picture naming paradigm disrupts speech production, in the form of speech arrest (anarthria), articulation errors (dysarthria) and phonological paraphasia (Fridriksson *et al.*, 2018). Recent studies have demonstrated that nTMS may correspond to DES when mapping posterior inferior frontal regions (pars opercularis, pars triangularis, ventral precentral cortex) well known to be essential in articulatory rehearsal, phonological processing and motor aspects of speech production (Dronkers *et al.*, 2007; Picht *et al.*, 2013; Tarapore *et al.*, 2013). Speech production errors are also commonly identified when stimulating the inferior parietal lobule, including the supramarginal gyrus and angular gyrus (Geschwind, 1965; Chang *et al.*, 2015; Igelström *et al.*, 2015; Sarubbo *et al.*, 2020). While errors can be induced across other perisylvian regions, correspondence between DES and nTMS has not been so clear-cut (Picht *et al.*, 2013; Tarapore *et al.*, 2013). Using diffusion tensor imaging in healthy subjects, it has been demonstrated that nTMS mapping using a picture naming paradigm is a reliable technique for identifying cortical terminations of the arcuate fasciculus, the core circuit of the wider language network (Catani *et al.*, 2005; Lin *et al.*, 2017). The arcuate fasciculus is made up of direct fronto-temporal connections (the long segment), and has also indirect connections with the inferior parietal lobule via the anterior segment (fronto-parietal) and posterior segment (parieto-temporal) (Catani *et al.*, 2005). As there is an indication for preoperative mapping and awake surgery when lesions are in proximity of the arcuate fasciculus (Sanai and Berger, 2008), it is relevant to determine whether the result reported in healthy subjects can be reproduced also in patients with heterogeneous brain lesions, particularly when a previous surgery has altered brain tissue in language-relevant regions (Duffau, 2014; Herbet *et al.*, 2016).

Diffusion imaging tractography has enabled indirect non-invasive mapping of white matter tracts involved in language function, using models to estimate the diffusion of water molecules (Basser *et al.*, 2000). Most commonly, nTMS stimulation sites are used as seed region-of-interests (ROIs) to track the arcuate fasciculus in tumour patients (Raffa *et al.*, 2016). However this does not take into account the entire structure of the arcuate fasciculus, nor its interindividual variability, relevant particularly in situations of heavily distorted anatomy due to the presence of a lesion. Furthermore, most studies combine nTMS with tensor-based modelling, which cannot correctly model fibre crossing within a single voxel (Dell’Acqua and Tournier, 2018). High angular resolution diffusion imaging models such as spherical deconvolution can overcome this limitation, even using clinical sequences with relatively low *b*-values, improving modelling of fibre crossing that is present in over 80% of white matter in the human brain (Dell’Acqua *et al.*, 2010; Farquharson *et al.*, 2013). We used nTMS and whole brain spherical deconvolution tractography in a broad spectrum of patients with brain tumours in perisylvian regions, to evaluate whether there is reliable correspondence between error sites identified with nTMS mapping during picture naming, and cortical terminations of the arcuate fasciculus.

## Materials and methods

### Participants

The study design was a prospective case collection. Patients scheduled for tumour removal in the vicinity of eloquent language-related areas were screened for enrolment, recruited from the Charité Hospital, Berlin between March 2013 and December 2016. Right-handed patients aged over 18 years were included, for whom there was the presence of a left hemisphere brain tumour compressing/infiltrating perisylvian cortical and subcortical language-related areas. Exclusion criteria were (i) moderate to severe aphasia, assessed on Aachen Aphasia Test, (ii) dementia (DemTect over 2), (iii) no planned surgery (iv) no good quality diffusion imaging acquisition. All patients provided written informed consent. The study proposal is in accordance with ethical standards of the Declaration of Helsinki and was approved by the local Ethics Commission.

### Image acquisition for nTMS and diffusion tractography

Datasets were acquired on a 3T Siemens scanner with an 8 channel head coil. T_1_-weighted 3D MPRAGE images were acquired for nTMS using the following parameters (echo train length: 1, TE: 2.67 ms, TR: 2.000, matrix size: 256 × 246, slice thickness: 1 mm). T_2_-weighted, FLAIR images were acquired to identify the non-contrast enhancing lesions (i.e. lower grade gliomas), using T_2_-weighted inversion recovery fast spin echo sequences (TR 6000 ms, TE 150 ms, TI 2000 ms). A diffusion weighted single-shot echo-planar sequence was used along 40 geometric directions with a *b*-value of 1000 s/mm^2^. One non-diffusion-weighted volume was collected. A matrix size of 128 × 128 was used, with a field of view of 240 mm × 240mm, and 2 mm isotropic voxels, with a TE of 83 ms and TR of 11 ms. Sixty contiguous 2 mm thick axial sections were acquired resulting in 2460 images.

### Navigated transcranial magnetic stimulation

#### Experimental setup

All patients underwent repetitive nTMS for localization of language-eloquent cortex. Mapping of both hemispheres was performed using the Nexstim NBS 4.3 with a NexSpeech module (Nexstim Oy, Helsinki, Finland) with particular focus around the peritumoural area and the regions surrounding the sylvian fissure (perisylvian) in the left hemisphere. The crus of the helix bilaterally, the nation and nine more scalp regions were used as cranial landmarks so that the system could co-register the patient’s head with the previously performed MRI scan. This enabled MR-guided targeting of specific regions with the coil, using the native anatomy of the patient. The maximum stereotactic error allowed by the co-registration software was 2 mm. Detailed information about the stimulation techniques and parameters have been described in previous studies (Krieg *et al.*, 2017). Briefly, the resting motor threshold (RMT) for the first dorsal interosseous was determined to identify the lowest cortical excitability threshold, which was applied at all stimulation points.

Stimulation was performed using a cooled figure-of-eight coil with an outer diameter of 16.5 cm and inner diameter of 9 cm. The coil was randomly moved in about 10 mm steps over the peritumoural and perisylvian cortex. The coil was oriented to be aligned perpendicular to the nearest sulcus, using real-time optical tracking to navigate it relative to each individual patient’s sulcal morphology, posterior to the stimulated point to achieve maximum field induction.

#### Speech mapping procedure

**Figure 1:**
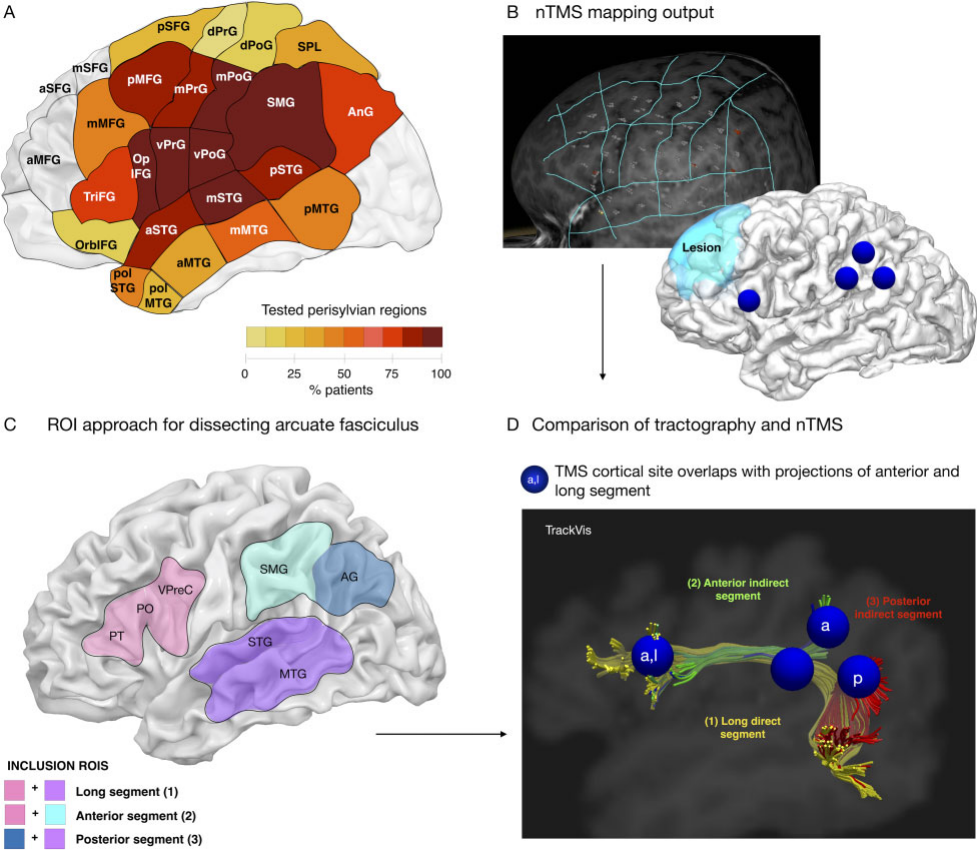
**Approach for combining TMS and tractography.** (**A**) The extent of testing in each left hemisphere region as a proportion of total patients, shown in relevant cortical areas [parcellation based on Corina *et al.* (2010)]. nTMS error sites are recorded in the Nexstim system (**B**) and exported. Whole brain spherical deconvolution of the arcuate fasciculus is performed using a two-ROI approach (**C**) and the error sites and tractography are compared in the native space of each patient using (**D**) Trackvis software.

An object picture naming paradigm was used during nTMS mapping, approved for clinical use according to Food and Drug Administration regulations, which is composed of black-and-white standardized line drawings of familiar objects, presented on a screen located in front of the patient. Patients underwent a baseline naming task three times, shown up to 150 objects (depending on clinical time constraints). During this, any objects which were unfamiliar, unrecognized or misnamed were eliminated to reduce false positive results as far as possible. The final number of objects presented to patients during the test session, and the number of baseline errors is reported in Table 1. Pictures were presented to the patient for 1 s, with individualized inter-picture interval of 2.5–4 s, depending on their performance in the baseline task. The words in English and German, along with their lexical characteristics are presented in Supplementary Table 1. According to the most recent literature, the repetitive nTMS stimulation was triggered alongside picture presentation, using an onset delay of 0 ms (Krieg *et al.*, 2017). The stimulation protocol comprised a train-of-five pulses with a 5 Hz frequency. The starting intensity was always 100% of RMT, however in certain patients, the intensity was increased in steps of 5% up to 130% of RMT if no language errors could be obtained in perisylvian areas, or the intensity could be decreased in steps of 5 if pain was reported, to avoid discomfort interfering with responses (Table 1). In case of negative mapping (no errors), stimulation intensity was increased and/or frequency increased to 7 pulses at 7 Hz, according to the protocol established by an expert panel for clinical nTMS mapping (Table 1; Krieg *et al.*, 2017). Patients were asked to express discomfort during the mapping through the Visual Analogue Scale from zero (no pain) to 10 (maximum pain). Stimulation intensity was reduced if patients complained about pain during the procedure. During the mapping procedure, 80–120 sites of the frontal, temporal and parietal cortex were stimulated at least three times each on non-consecutive trials (Fig. 1A). An error site was recorded when at least one of the three stimulations produced a speech error. Even if there were more than one errors at a single site this was counted only once. Stimulation was performed over both healthy tissue and the tumour. The software automatically registered the location of each stimulation spot. The coil was not kept at the same site for consecutive stimulation trains, to avoid summation effects.

**Table 1:** Demographic information on patient group and individual parameters for transcranial magnetic stimulation language mapping

	Patient no.	Age	Sex	Handedness	Histology	Tumour location	Tumour volume (cm^3^)	Cavity volume (cm^3^)	RMT (% of maximum stimulator output)	Proportion of RMT used for task (%)	Stimulation intensity	Total number of pictures	Baseline errors	Total no. stimulations	Total no. naming errors	Stimulation frequency
First surgery	1	38	M	R	Glioma (WHO III)	Fr	5.5		36	90	32	78	2/80	221	3	5 Hz/5 Stim
	2	49	F	L	Glioma (WHO III)	Fr	9.12		36	90	32	73	7/80	137	4	5 Hz/5 Stim
	3	55	F	R	Metastasis (breast carcinoma)	Fr	8.2		32	100	32	60	20/80	170	8	5 Hz/5 Stim
	4	29	M	R	Oligodendroglioma (WHO II)	Fr	40		28	100	28	66	14/80	214	17	5 Hz/5 Stim
	5	39	F	R	Glioma (WHO III)	TPO	18.63		34	100	34	65	15/80	216	15	5 Hz/5 Stim
	6	67	M	R	Glioma (WHO III)	AT	4.7		26	100	26	133	17/150	149	11	5 Hz/5 Stim
	7	40	M	R	Ependymoma (WHO II)	TPO	13.4		26	100	26	75	5/80	198	4	7 Hz/7 Stim
	8	28	F	R	Glioma (WHO III)	Fr	24.6		39	100	39	90	10/80	192	11	5 Hz/5 Stim
	9	51	F	R	Glioma (WHO III)	AT	45.53		35	90	31	128	22/150	189	4	5 Hz/5 Stim
	10	36	F	R	Astrocytoma (WHO II)	AT	10.2		49	70	34	77	3/80	231	5	5 Hz/5 Stim
	11	66	M	R	Metastasis (renal cell carcinoma)	AT	2.6		30	100	30	64	16/80	222	6	5 Hz/5 Stim
	12	69	F	R	Glioma (WHO IV)	AT	20.6		34	80	27	44	36/80	190	15	5 Hz/5 Stim
	13	60	M	R	Glioma (WHO IV)	Fr	8		36	100	36	46	50/96	169	6	5 Hz/5 Stim
	14	33	F	R	Glioma (WHO III)	Fr	28.1		29	100	29	72	8/80	228	3	5 Hz/5 Stim
	15	54	M	R	Glioma (WHO IV)	AT	3.3		43	120	52	128	22/150	207	12	7 Hz/7 Stim
	16	41	F	R	Glioma (WHO III)	Fr	0.65		37	90	33	101	17/118	167	3	5 Hz/5 Stim
Second surgery	17	51	F	R	Glioma (WHO IV)	Fr	8.9	8.5	32	100	32	68	12/80	174	4	5 Hz/5 Stim
	18	21	M	R	Glioma (WHO IV)	Fr	69.5	3.1	28	115	32	74	6/80	186	9	5 Hz/5 Stim
	19	32	M	R	Glioma (WHO III)	AT	59.8	0.9	30	130	39	76	4/80	194	8	5 Hz/5 Stim
	20	40	F	R	Oligoastrocytoma (WHO II)	AT	17.3	1.7	50	80	40	97	53/150	169	8	7 Hz/7 Stim
	21	34	M	L	Oligoastrocytoma (WHO III)	TPO	4.7	2.2	38	70	27	74	6/80	175	9	5 Hz/5 Stim
	22	35	M	R	Ganglioglioma (WHO I)	TPO	1.7	0.5	34	100	34	80	0/80	150	4	5 Hz/5 Stim
	23	50	F	R	Glioma (WHO III)	TPO	3.4	5.1	32	100	32	64	16/80	206	3	5 Hz/5 Stim
	24	37	M	R	Glioma (WHO III)	TPO	36.2	5.3	34	90	31	73	7/80	162	12	5 Hz/5 Stim
	25	77	M	R	Glioma (WHO IV)	AT	8	5.4	27	110	30	49	31/80	153	15	5 Hz/5 Stim
	26	31	F	R	Glioma (WHO III)	AT	6.23	4.7	30	100	30	113	37/150	146	6	5 Hz/5 Stim
	27	37	F	R	Oligoastrocytoma (WHO III)	TPO	6.4	3.3	24	100	24	58	22/80	177	10	5 Hz/5 Stim
	28	35	M	R	Glioma (WHO III)	AT	9.06	0.5	29	110	32	120	30/150	194	15	5 Hz/5 Stim

Sex: M, male; F, female; Handedness: R, right-handed; L, left-handed; Location of tumour: Fr, frontal; TPO, temporo-parieto-occipital; AT, anterior temporal, calculated as percentage of maximum stimulator output.

#### Data analysis

The whole procedure was analysed offline by a specialist neurolinguist. Errors included in the analysis were anarthria (a complete lack of response), dysarthria (form-based distortions that are slurred, stuttered or imprecisely articulated) and phonemic paraphasia (unintended phonemic modification of the target word, e.g. /pænts/is replaced with/fants/). We classed all of the above error types as speech articulation errors.

### Diffusion imaging processing and tractography dissection

Data were corrected for head motion, eddy current distortion and susceptibility artefacts using ExploreDTI. While tensor-based approaches are commonly recommended for sequences with low *b*-values (Jones *et al.*, 2013), a number of recent studies have indicated that spherical deconvolution techniques may also be used and provide fewer false negatives (Farquharson *et al.*, 2013; Calamuneri *et al.*, 2018). Spherical deconvolution tractography was hence calculated using StarTrack software (www.natbrainlab.co.uk), using a damped Richardson–Lucy algorithm (Dell’Acqua *et al.*, 2010). A fibre response parameter of alpha = 1.5, 200 algorithm iterations, and *n* = 0.15 and *v* = 15 as threshold and geometrical regularization parameters. An absolute and relative threshold was applied to exclude local spurious maxima. Whole brain deterministic tractography was used, using Euler interpolation to reconstruct streamlines (Dell’Acqua *et al.*, 2010). Spherical deconvolution tractography estimates multiple fibre directions within a voxel, constituting an advantage over diffusion tensor methods which cannot resolve multiple orientations within a voxel (Dell’Acqua and Tournier, 2018). When entering a region with crossing white matter bundles, the algorithm followed the orientation vector of least curvature. Streamlines were halted when a voxel without fibre orientation was reached or when the curvature between two steps exceeded a threshold of 45 degrees.

Tractography dissections were performed by the first authors (D.G. and H.H.), using a region-of-interest (ROI) based approach described in a previous study (Forkel *et al.*, 2014). A three-ROI approach was used on anisotropic power maps defined in the patient’s native space (Fig. 1C). The frontal ROI was defined on sagittal slices, anterior to the central sulcus to incorporate the posterior inferior frontal white matter (pars opercularis and pars triangularis) and ventral precentral gyrus. The parietal ROI was defined on sagittal slices to incorporate the white matter of the supramarginal gyrus and angular gyrus, and the temporal ROI was defined on sagittal and axial slices to incorporate the posterior superior and middle temporal gyrus. The direct long segment was defined as all streamlines running between the frontal and temporal ROI, while the indirect segments included those running between frontal and parietal ROIs (anterior segment) or parietal and temporal ROIs (posterior segment). This is in line with previous studies (Catani *et al.*, 2005). In certain cases where the pathology had altered anatomical landmarks used to define brain regions, the ROIs had to modified to reflect this.

### Comparison of TMS and tractography

Error sites were saved onto the patient’s T1 image used for navigation by the Nexstim software (Fig. 1B). Each site was anatomically localized on a standard parcellation template (Corina *et al.*, 2010) and checked by an expert anatomist (H.H.). The sites were registered to the anisotropic power maps (Chen *et al.*, 2019) and imported into the tractography software as 6 mm diameter spherical seed ROIs to cover size of the anatomical locus of stimulation (Romero *et al.*, 2019) for each patient using non-linear registration tools from the FMRIB Software Library (FSL, Jenkinson *et al.*, 2012). Stimulation sites where then adjusted at the grey-white matter boundary.

Each spherical ROI was overlaid and compared with the pre-traced segments of the arcuate fasciculus (Fig. 1D). Errors were attributed to four tract categories—anterior segment, long segment, posterior segment, non-arcuate, only if there was at least one overlapping voxel between the TMS ROI and the streamlines belonging to that tract. If error sites were in locations corresponding to streamlines belonging to more than one segment of the arcuate, they were classified in both (e.g. a site in inferior frontal regions could be recorded as corresponding to terminations of both the anterior and long segment). A number of error sites were identified within the tumour, however as tractography is commonly unreliable in tracking through lesioned tissue, we did not include these sites in the analysis comparing error sites with tractography reconstructions of the arcuate fasciculus.

### Statistical analysis

All statistical analysis was performed using SPSS (version 26). Linear regressions were performed to identify whether demographics (age, sex or handedness), clinical characteristics (tumour volume, tumour location, resection volume or grade), TMS variables (RMT, number of total stimulations or proportion of baseline errors) or tract measurements were linked to the number of speech errors made. Wilcoxon signed-rank tests were conducted to assess which branches of the arcuate fasciculus were more linked to error sites than others, and how this differed between lobes.

### Data availability statement

The spreadsheets that support the findings of this study are available on the Open Science Framework (https://osf.io/xyekp/). The clinical data is available on reasonable request to the first or last author (D.G. or T.P.).

## Results

### Participants

**Figure 2:**
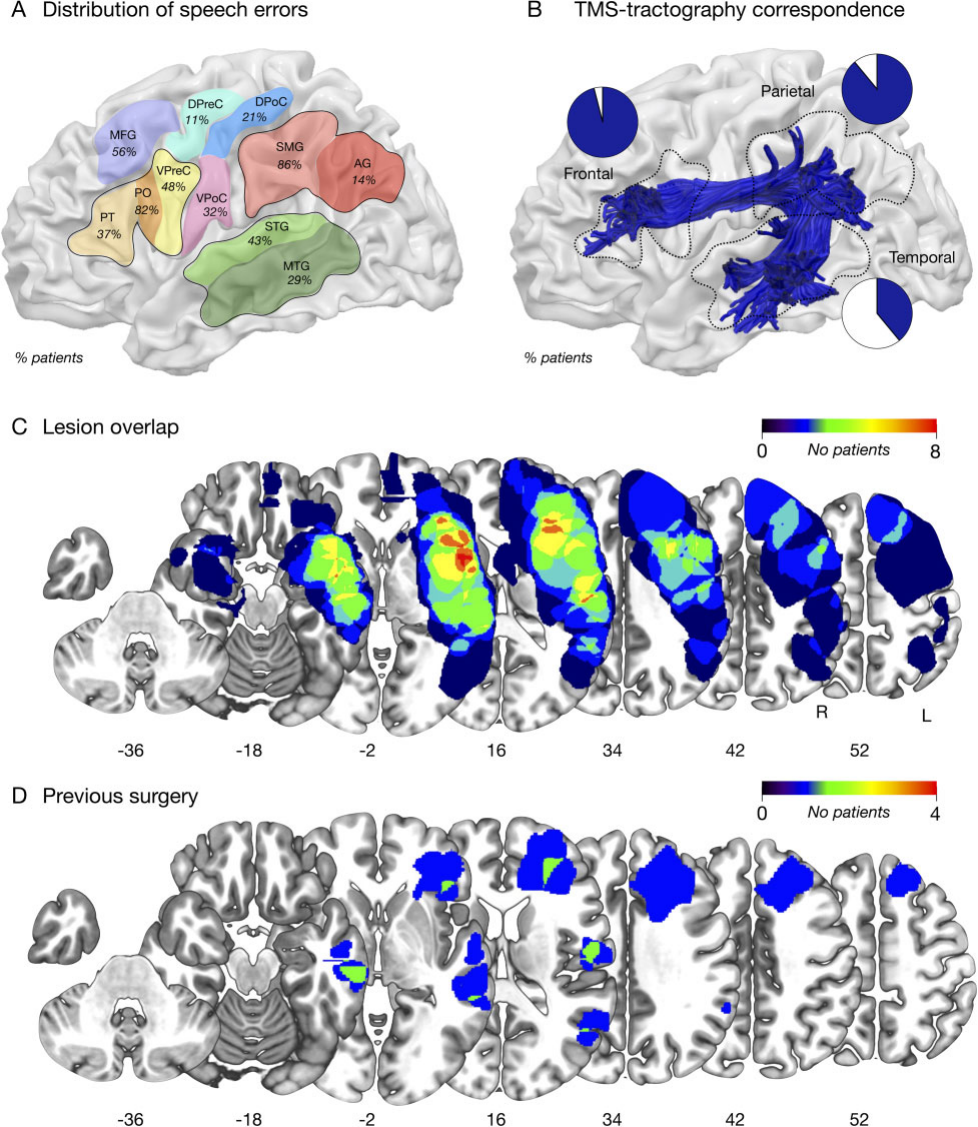
**Correspondence between TMS and tractography.** (**A**) The proportion of patients in which speech errors were induced by TMS in different cortical perisylvian sectors. (**B**) The proportion of patients with an error site that corresponded to a cortical termination of the entire arcuate fasciculus (shown on a 3D standard space template in blue), within each lobe. (**C**) Percentage overlay map of the tumour locations in left hemisphere perisylvian regions. (**D**) Percentage overlay map of the previous resections in left hemisphere perisylvian regions.

Thirty patients were considered for the study, of which two patients were excluded as two or fewer speech errors could be induced (14 male, mean age 44 SD 14; Table 1). The majority of patients were undergoing surgery for a glioma with a grade of III or IV. Sixteen patients underwent nTMS mapping for a first surgery, and 12 patients were returning for a second surgery. In the entire sample (Fig. 2C), 10 patients had a frontal lobe lesion, 7 patients had a temporo-parieto-occipital lesion, 11 had a temporal lesion. Mean tumour volume was 16.9 cm^3^ (SD 17.8). The mean previous resection cavity size in the 12 returning patients was 3.4 cm^3^ (SD 2.4; Fig. 2D). No patients reported discomfort during the nTMS procedure and no adverse events occurred (e.g. seizures).

### Cortical localization of nTMS speech errors

The perisylvian cortex was mapped using nTMS in the left hemisphere of all 28 patients (mean number of stimulations 185, SD 26.5; Fig. 1A). The RMT and the proportion of baseline errors for each patient are reported in Table 1. We show all the error sites identified in each patient and their anatomical location in Supplementary Figs 1 and 2. We first performed linear regressions to identify interactions between demographic, clinical or TMS variables with the number of speech errors induced. This showed no significant interactions, including no influence of previous surgery on the result. The number of baseline errors approached significance [*F*(1,26)=4.1, *P* = 0.052].

We first examined the prevalence of speech errors in different cortical areas across the entire group (Fig. 2A). The mean number of errors was 8 per patient (SD 4.5). At least one error was recorded in all patients when stimulating the frontal region (100%). Errors were identified in 92% of patients when stimulating the parietal region, and 53% of patients in the posterior temporal region.

We next compared the prevalence of errors within each lobe. Within the frontal lobe, stimulation of the pars opercularis produced errors most frequently (82% of patients). Errors were also identified in around half of patients in the middle frontal gyrus (56%) and ventral precentral gyrus (48%). Errors were also identified to a lesser extent in the pars triangularis (37%) and a more dorsal region of the precentral gyrus closer to the hand representation (11%). In the parietal lobe, stimulation of the supramarginal gyrus resulted in at least one picture naming error in 86% of patients, and occurred less commonly when stimulating the angular gyrus (14%). Stimulation of the postcentral gyrus also produced errors, in 32% of patients when stimulating ventral postcentral regions and 21% of patients in a more dorsal region closer to the hand representation. In the temporal lobe, at least one error was identified in the superior temporal gyrus in 43% of patients, and the middle temporal gyrus in 29% of patients.

### Comparison of nTMS speech errors and cortical projections of the arcuate fasciculus

We examined if error sites were significantly linked with a projection of the arcuate fasciculus within each lobe, excluding those sites identified on tumorous tissue (Fig. 2B). Wilcoxon signed-rank tests showed that in the parietal lobe, there were significantly more error sites associated with a cortical projection of the arcuate than not (*Z* = −4.3, *P* < 0.001), and this approached significance also in the frontal lobe (*Z* = −1.9, *P* = 0.05). This was not significant in the temporal lobe (*Z* = −4.6, *P* = 0.6).

We examined whether the individual stimulation sites corresponded to a termination of the patient’s arcuate fasciculus dissected with tractography (the anterior, posterior and long segments). We report tract measurements for all three segments in Table 2 (mean arcuate volume 18 ml, SD 7.7). The anterior segment (mean 5.9 ml SD 3.1) could be reconstructed in all but three patients, and the long segment (7.2 ml SD 3.3) was missing in one patient. The posterior segment (5.9 ml SD 3.5) was also missing in one patient. We first performed regressions to evaluate whether any clinical or demographic variables were associated with tract volume for any of the three arcuate segments, or the entire fascicle. This showed no effect of any of these variables on tract volume, including no influence of the previous surgery.

**Table 2:** Tract measurements for the arcuate fasciculus in the left hemisphere

Patient number	Group	Anterior segment volume (ml)	Long segment volume (ml)	Posterior segment volume (ml)	Entire arcuate volume (ml)
1	No previous surgery	8.18	12.66	9.84	30.7
2	No previous surgery	2.67	8.06	3.89	14.6
3	No previous surgery	2.72	4.37	6.15	13.2
4	No previous surgery	2.37	3.07	5.72	11.2
5	No previous surgery	4.24	7.14	3.28	14.7
6	No previous surgery	9.29	5.47	1.24	16.0
7	No previous surgery	8.14	7.58	2.78	18.5
8	No previous surgery			5.18	5.2
9	No previous surgery	8.73	10.81	8.51	28.1
10	No previous surgery	4.05	8.59	2.92	15.6
11	No previous surgery	4.60	7.37	5.16	17.1
12	No previous surgery	10.09	7.93	9.10	27.1
13	No previous surgery	2.90	13.74	7.70	24.3
14	No previous surgery		2.69	9.15	11.8
15	No previous surgery		3.92	0.56	4.5
16	No previous surgery	0.97	8.65	12.98	22.6
17	Previous surgery	3.38	3.95	7.06	14.4
18	Previous surgery	3.24	10.10	7.18	20.5
19	Previous surgery	3.52	5.71	7.00	16.2
20	Previous surgery	6.74	8.10	2.82	17.7
21	Previous surgery	7.88	2.24		10.1
22	Previous surgery	12.80	12.41	14.47	39.7
23	Previous surgery	9.06	8.41	8.65	26.1
24	Previous surgery	2.81	6.12	6.03	15.0
25	Previous surgery	7.71	8.09	1.20	17.0
26	Previous surgery	7.24	2.45	6.15	15.8
27	Previous surgery	8.43	11.17	3.26	22.9
28	Previous surgery	4.99	2.80	2.16	10.0

**Figure 3:**
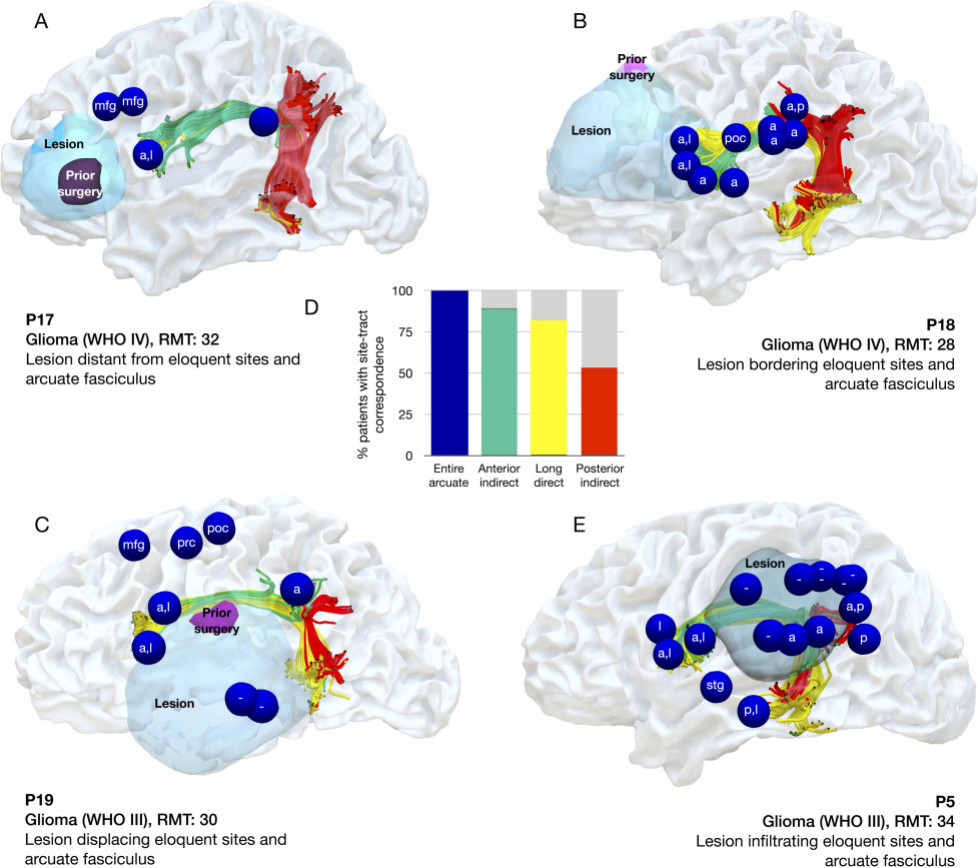
**Four patient examples of correspondence between TMS and tractography.** The distribution of errors sites (blue spheres), the lesion (light blue) or previous surgery (purple) and the individual arcuate fasciculus (red, yellow, green). (**A**) In Patient 17 the lesion is located distantly from the arcuate fasciculus and the TMS-induced speech error sites. (**B**) In patient 18 the lesion is bordering the arcuate fasciculus in the frontal lobe. (**C**) In patient 19, the arcuate fasciculus is displaced by the lesion. (**D**) Bar graph showing the proportion of patients with a site that corresponds to the entire arcuate fasciculus or one segment. (**E**) In patient 5, the lesion has infiltrated the arcuate fasciculus in the parietal lobe. Tracts: A, anterior segment; l, long segment; p, posterior segment of the arcuate fasciculus. A dash refers to a site located on the tumour that was not associated with the arcuate fasciculus. Cortical sites: mfg, middle frontal gyrus; poc, postcentral; stg, superior temporal gyrus; smg, supramarginal gyrus.

Our results clearly showed that at least one error site corresponded to a cortical termination of the arcuate fasciculus in every patient, even in cases where the arcuate had been considerably displaced or infiltrated (Fig. 3; Supplementary Figs 1 and 2). A Wilcoxon signed-rank test showed there were significantly more error sites associated with arcuate projections than those not associated with the arcuate (*Z* = −3.6, *P* = 0.001). In particular, error sites corresponded to cortical projections of the anterior segment in 90% of patients, with the long segment in 82% of patients and the posterior segment in 53% of patients (Fig. 3D). Wilcoxon signed-rank tests showed significantly more error sites associated with the anterior segment than the long segment (*Z* = −3.7, *P* < 0.001) and posterior segment (*Z* = −4.2, *P* < 0.001), and significantly more sites associated with the long segment than the posterior segment (*Z* = −2.7, *P* = 0.006).

## Discussion

In this study, we evaluated whether nTMS is a reliable tool for mapping the functional architecture of the arcuate fasciculus in a heterogenous cohort of patients with lesions in perisylvian regions. We examined whether, as previously described in healthy subjects (Lin *et al.*, 2017), there was good correspondence between nTMS-induced speech errors and the arcuate fasciculus in patients with altered white matter anatomy due to a lesion, and/or a previous surgery. We showed that nTMS speech errors, including anarthria, dysarthria and phonological paraphasias, occur most commonly when stimulating frontal and parietal regions and corresponded in all cases with at least one anatomical termination of the arcuate fasciculus. This result was evident in both patients undergoing a first surgery in a perisylvian area, and those awaiting a second surgery. Our preliminary results indicate that the underlying white matter anatomy may be a good indicator of the migration of functionally eloquent cortex, which is of great relevance to presurgical planning. As this was consistent in both first and second surgery groups, even with considerable infiltration/displacement of brain tissue, we suggest that integrating nTMS mapping using picture naming and diffusion tractography into the presurgical plan may be a useful tool to evaluate functional architecture in patients with perisylvian brain lesions.

### nTMS speech mapping is effective in identifying eloquent frontal and parietal regions

We show that speech error sites induced by nTMS are reliable for mapping the arcuate fasciculus in its entirety, and particularly when targeting the pars opercularis in the frontal lobe and the supramarginal gyrus in the parietal lobe. A previous study (Lin *et al.*, 2017) using a similar approach in healthy controls (nTMS and tractography, a picture naming paradigm and *n* = 28), also showed that the inferior frontal gyrus could reliably elicit picture naming errors with TMS, although they did not find similar results in the parietal lobe. However, when comparing TMS results with the underlying white matter anatomy (Lin *et al.*, 2017) also showed very good correspondence between error sites in both the frontal and parietal lobe, and cortical terminations of the arcuate fasciculus. While caution needs to be taken in comparing our results with this study as the nTMS and tractography methodologies are not exactly analogous, these results may be clinically useful.

Diffusion tractography of the arcuate may be a robust marker of the location of essential functional cortex, hence integrating a reliable tractography reconstruction into the MR-navigation system prior to language mapping may improve mapping specificity. Stimulation studies using DES as well as nTMS have shown that language function can reorganize in patients awaiting a second operation, with function potentially moving from the operated area (Krieg *et al.*, 2014; Southwell *et al.*, 2016). In our study, all patients showed errors involving the arcuate fasciculus, irrespective of any clinical features. This result was robust, even when the tumour had quite dramatically altered the anatomy. Our results indicate that even when a previous surgery in a perisylvian area has been performed, the arcuate fasciculus appears to be a reliable signature of the migration of functionally eloquent cortex.

Our results indicate that nTMS mapping particularly identified cortical terminations of different segments of the arcuate fasciculus. In fact, while the arcuate fasciculus was traditionally described solely in its fronto-temporal component (Geschwind, 1965), more recently it has been shown to have parietal ‘indirect’ projections which have been incorporated into modern models of language organization (Catani and Bambini, 2014; Tremblay and Dick, 2016; Poeppel and Assaneo, 2020). Interindividual differences in segments of the arcuate fasciculus have been linked to differences in language ability in a number of studies (Forkel *et al.*, 2020*a*, *b*). Terminations of the fronto-parietal anterior segment (also termed the superior longitudinal fascicle III) were identified with nTMS almost 90% of patients. This tract plays a direct role in motor programming for articulation, as well as in short-term memory for phoneme articulation, which is supported by intraoperative studies (Papagno *et al.*, 2017). DES of the anterior segment reliably induces dysarthria and speech arrest both at its cortical terminations in the frontal and parietal lobe (Duffau *et al.*, 2003) as well as after subcortical stimulation (van Geemen *et al.*, 2014). A recent study of 256 patients undergoing awake surgery described how cortical and subcortical stimulation of the anterior segment using DES causes interference particularly with speech articulation and speech output over other facets of language, for example reading and non-verbal comprehension (Sarubbo *et al.*, 2020). Lesion studies also show that disconnection of the anterior segment over other arcuate segments is linked to speech production difficulties following stroke (Fridriksson *et al.*, 2013) and damage to this tract is associated with apraxia of speech (Fridriksson *et al.*, 2018). Previous studies have shown that DES applied over the pars opercularis prevents speech production without affecting oro-facial and tongue muscle activity, whereas stimulation over ventral precentral gyrus does not: here there is interference with basic phono-articulatory muscle activity (Cerri *et al.*, 2015; Ferpozzi *et al.*, 2018). Future studies integrating electromyography may be able to dissociate these different aspects also when using TMS. Given this tract is also relevant in sensorimotor integration for goal-directed actions of the upper limb, its identification and preservation in preoperative planning may have wider functional impact than just for speech articulation (Howells *et al.*, 2018).

Speech error sites also corresponded to cortical terminations of the direct fronto-temporal segment of the arcuate fasciculus (the long segment) in around 80% of patients. This tract is the major component of the language circuit, dedicated to mapping verbal input onto articulatory-based representations, including phonological elaboration and encoding (Sarubbo *et al.*, 2015; Tremblay and Dick, 2016) and processing of syntactically complex sentences (Vidorreta *et al.*, 2011; Poeppel and Assaneo, 2020). Intraoperative stimulation of the long segment accurately and reliably induces phonemic paraphasias (Bello *et al.*, 2007; Benzagmout *et al.*, 2007). Furthermore, it mediates fast interaction between auditory and motor areas, thus facilitating phoneme categorization to create motor codes of the new phonological sequences, i.e. word learning (Lopez-Barroso *et al.*, 2013). Our results indicated that the TMS protocol used was reliable in identifying its cortical terminations when in the frontal lobe, as far fewer speech errors could be elicited in the temporal lobe.

Error sites also corresponded to terminations of the posterior ‘indirect’ segment of the arcuate fasciculus in around half of the patient group, connecting the parietal and temporal lobes. This rate may have been lower as speech errors could not be elicited so commonly in the temporal lobe (also identified in around half of patients). As a recent language model has indicated that the temporo-parietal segment of the arcuate fasciculus plays an important role in speech production, specifically in word repetition, we propose that this lower incidence of errors may be due to the paradigm used (Forkel et al., 2020*a*). To name a picture, one must recognize the object, categorize it semantically, retrieve and select the target word and phonologically encode this, thus converting incoming visual information into articulatory output. However, in models of language, the posterior temporal lobe is classically associated with auditory processing (Indefrey and Levelt, 2004; Hickok and Poeppel, 2007). Thus, the lower number of errors identified in the temporal lobe may be a reflection of the behavioural paradigm used: were the cognitive load on auditory processing increased, nTMS may be more effective in preoperative mapping also in the temporal lobe. This may be achieved by implementing verb or noun generation tasks, repetition, acoustic or reading paradigms into nTMS, which are used in intraoperative mapping for identification temporal sites (Duffau *et al.*, 2014; Sarubbo *et al.*, 2020). Future studies may be able to determine the underlying mechanism limiting the efficiency of nTMS in mapping the temporal lobe.

Another aspect for discussion is the error sites identified that were not associated with the arcuate fasciculus. These were identified in the precentral, postcentral and middle frontal gyri. Direct motor responses for face muscles from both the pre- and post-central gyri are well established (Penfield and Boldrey, 1937). Moreover, errors in ventral regions of the postcentral gyrus are likely related to disruption of facial sensorimotor synergies, as tightly linked interactions between precentral and postcentral cortices are crucial during syllabification (Bouchard *et al.*, 2013). The middle frontal gyrus, and particularly its most posterior part, is involved in semantic aspects of language processing, as well as switching between languages (Binder *et al.*, 2009; Sierpowska *et al.*, 2018): hence it is possible that stimulation here interfered with the broader language network. Alternatively, this result could be explained as a disruption of the attentional network, targeting the frontal eye fields connected to the parietal lobe via the middle branch of the superior longitudinal fasciculus II, which runs in parallel to the anterior segment (or superior longitudinal fascicle III). There were also considerable speech errors identified in tumorous or perilesional areas: in these regions tractography can be limited in tracing white matter tracts due to oedema and partial volume effects (Catani and Dell’Acqua, 2011). New diffusion imaging techniques such as three-tissue constrained spherical deconvolution have been developed that may be better able to track through lesioned tissue. However, mapping of eloquent tissue within and surrounding the tumour is particularly critical, thus DES is evidently still required within the surgical routine and our results indicate that preoperative techniques should still be considered adjunctive to the gold standard rather than a replacement.

### nTMS-tractography and intraoperative mapping

nTMS has certain advantages over DES: it is non-invasive therefore is not restricted to the area of the craniotomy, and can support presurgical planning (Picht *et al.*, 2013). It is also safe and well tolerated, with a very low seizure risk (Tarapore *et al.*, 2016). In our study, we show that induced speech errors during nTMS mapping with picture naming corresponded to the arcuate fasciculus in all patients, particularly the fronto-parietal anterior segment (or superior longitudinal fascicle III). Speech errors occurred at both frontal and parietal cortical terminations in patients with the anterior segment, while in patients without no parietal site was identified. The protocol followed in this study used guidelines advised by an expert clinical panel for the presurgical application of nTMS (Krieg *et al.*, 2017). This indicates that nTMS and tractography are highly complementary methods and are sensitive to interindividual variability, which was remarkably evident in our heterogeneous patient group. This is highly relevant when combining structural and functional information for preoperative planning, where tissue is damaged. We did not have access to the intraoperative stimulation sites identified in these patients, however it would be of value for future studies to evaluate whether the positive predictive value of nTMS for DES improves if targeted on the fronto-parietal branch of the arcuate using this paradigm, or can detect errors more widely if changing the paradigm to include auditory feedback.

## Limitations and conclusions

There are several limitations to our study. The tractography acquisition used was a common clinical acquisition using a relatively low *b*-value (1000), and there is some discrepancy in the literature as to whether this can be used for higher quality reconstructions than those made possible with the diffusion tensor (Jones, 2010; Calamuneri *et al.*, 2018). As our diffusion sequence was not up to the standards commonly used in research protocols, it is possible that we were unable to reconstruct streamlines projecting to other positive sites highlighted by TMS (false negatives). Future studies using more advanced sequences may be able to determine whether this is the case. Moreover, our sample size was relatively limited and therefore this should be considered preliminary. Further studies may confirm our results in a larger cohort. As speech errors are relatively rare per subject, we grouped different types of speech articulation error to improve power, however in a larger cohort it would be possible to evaluate whether there were specific pattern at cortical sites. Furthermore, the association between the number of baseline errors and the number of errors during mapping approached significance, which may indicate that the patient’s pre-existing language deficits may contribute to the number of errors identified during stimulation. We also did not perform repeated TMS mappings in the same subject, but it would be important to do this to evaluate individual reproducibility of the results. We cannot also rule out the role of other white matter tracts that have been linked to speech output such as the frontal aslant tract, linking the inferior and superior frontal gyri. Picture naming is also just one of many aspects of language that may be important to test preoperatively, such as spoken word recognition, syntax or sentence understanding (Ohlerth *et al.*, 2020). Furthermore, it is not possible to evaluate the precise relationship between the cortex and the streamline endings using tractography due to partial volume effects at the grey–white matter boundary. However, given the wide field of TMS, we judged it possible to estimate likely white matter streamlines by extending the radius of the identified site (3 mm) into white matter.

To conclude, we show that preoperative nTMS mapping in a heterogeneous cohort of patients with brain tumours is reliable in causing interference in speech production during picture naming, specifically at sites corresponding to both frontal and parietal terminations of the arcuate fasciculus, termed the anterior segment or ventral branch of the superior longitudinal fasciculus III. Furthermore, we show that nTMS mapping is robust in identifying these terminations, even when white matter is displaced or infiltrated by the lesion, or a previous surgery has taken place. As there was lower reliability for mapping the temporal projections of the arcuate fasciculus, and given previous literature indicating a lesser role for these tracts in speech production *per se*, we propose that altering the behavioural paradigm may improve the positive predictive value of nTMS for DES, and preoperative mapping. Our results indicate that a combined functional–anatomical approach may be a relevant tool in providing personalized targets for DES in mapping speech production, or alternative choices of surgical strategy.

## Supplementary material

Supplementary material is available at *Brain Communications* online.

## Supplementary Material

fcaa158_Supplementary_Data

## Abbreviations

DES =: direct electrical stimulation;

nTMS =: navigated transcranial magnetic stimulation;

RMT =: resting motor threshold;

ROI =: region-of-interest;

TMS =: transcranial magnetic stimulation.
