# Supplementary material for: Preoperative transcranial magnetic stimulation for picture naming is reliable in mapping segments of the arcuate fasciculus
Source: Brain Commun. 2020 Sep 29;2(2):fcaa158. doi: 10.1093/braincomms/fcaa158 (PMC7846168; doi:10.1093/braincomms/fcaa158)
Supplement: fcaa158_Supplementary_Data [file fcaa158_supplementary_data.pdf]

Supplementary Information

Supplementary Figure 1. Subset of patients undergoing first surgery (a) Overlap of patient tumours (b) Individual patient transcranial magnetic stimulation (TMS) sites and tractography of the arcuate fasciculus.

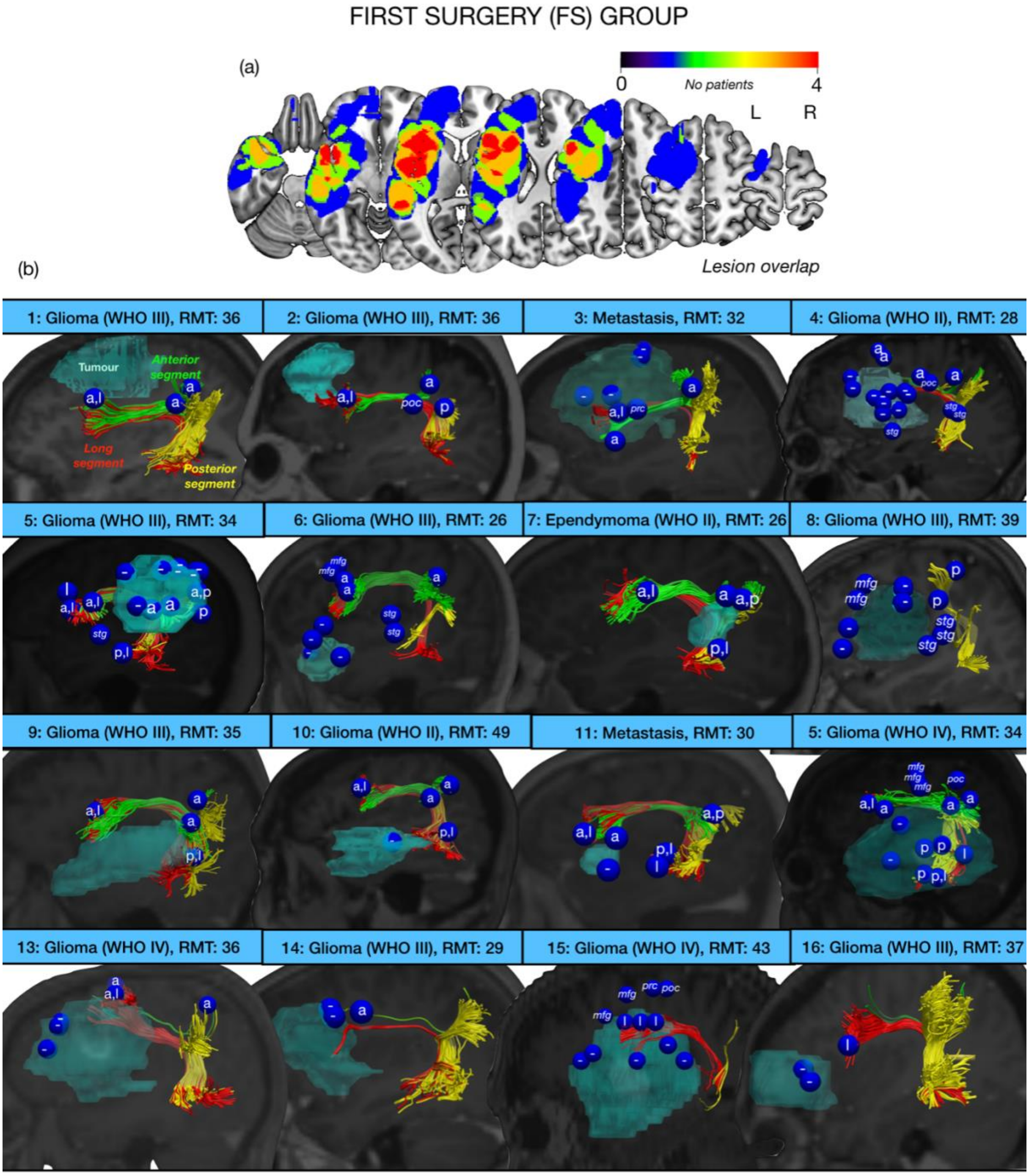

Supplementary Figure 2. Subset of patients undergoing second surgery. Top: overlap of patient resection cavities. Blue panels: individual patient transcranial magnetic stimulation (TMS) sites and tractography of the arcuate fasciculus.

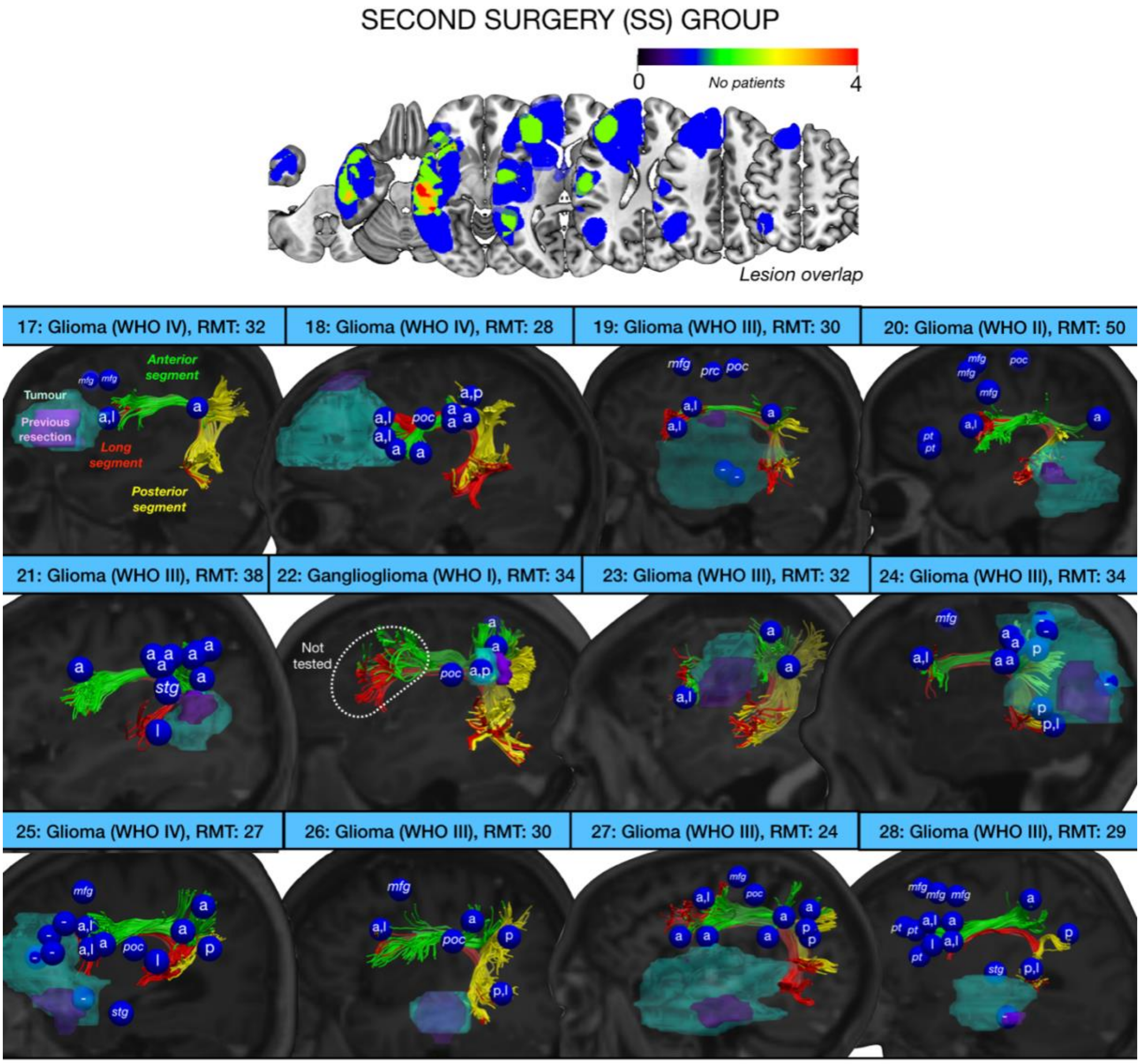

Supplementary Table 1. Lexical characteristics of target object words.

Number	Target word in English	Target word in German	Number of letters	Number of syllables
1	Airplane	Flugzeug	8	2
2	Apple	Apfel	5	2
3	Arrow	Pfeil	5	1
4	Artichoke	Artischocke	11	4
5	Ax	Axt	3	1
6	Baby	Baby	4	1
7	Backpack	Rucksack	8	2

8	Bag	Tüte	4	2
9	Ball	Ball	4	1
10	Ballon	Luftballon	10	3
11	Banana	Banane	6	3
12	Barrel	Fass	4	1
13	Basket	Korb	4	1
14	Bell	Glocke	6	2
15	Belt	Gürtel	6	2
16	Bench	Bank	4	1
17	Bicycle	Fahrrad	7	2
18	Binoculars	Fernglas	8	2
19	Bird	Vogel	5	2
20	Boat	Boot	4	1
21	Bomb	Bombe	5	2
22	Bone	Knochen	7	2
23	Book	Buch	4	1
24	Boot	Stiefel	7	2
25	Bottle	Flasche	7	2
26	Bowl	Schüssel	8	2
27	Box	Karton	6	2
28	Boy	Junge	5	2
29	Bread	Brot	4	1
30	Brush	Bürste	6	2
31	Bucket	Eimer	5	2
32	Butter	Butter	6	2
33	Butterfly	Schmetterling	13	3
34	Button	Knopf	5	1
35	Cake	Kuchen	6	2
36	Camera	Kamera	6	3
37	Candle	Kerze	5	2
38	Car	Auto	4	2
39	Carrot	Mohrrübe	8	3
40	Cat	Katze	5	2
41	Chair	Stuhl	5	1
42	Cheese	Käse	4	2
43	Cigarette	Zigarette	9	4
44	Clock	Uhr	3	1
45	Clothespin	Wäscheklammer	13	4
46	Cookie	Keks	4	1

47	Corn	Mais	4	1
48	Dime	Geld	4	1
49	Dog	Hund	4	1
50	Doll	Puppe	5	2
51	Dress	Kleid	5	1
52	Drum	Trommel	7	2
53	Ear	Ohr	3	1
54	Egg	Ei	2	1
55	Envelope	Briefumschlag	13	3
56	Fan	Ventilator	10	4
57	Faucet	Wasserhahn	10	3
58	Feather	Feder	5	2
59	Fence	Zaun	4	1
60	Fire	Feuer	5	2
61	Flashlight	Taschenlampe	12	4
62	Flower	Blume	5	2
63	Flute	Flöte	5	2
64	Foot	Fuß	3	1
65	Football	Fußball	7	2
66	Fork	Gabel	5	2
67	Frog	Frosch	6	1
68	Funnel	Trichter	8	2
69	Glass	Glas	4	1
70	Glasses	Brille	6	2
71	Globe	Globus	6	2
72	Glove	Handschuh	9	2
73	Guitar	Gitarre	7	3
74	Hamburger	Hamburger	9	3
75	Hammer	Hammer	6	2
76	Hand	Hand	4	1
77	Handcuffs	Handschellen	12	3
78	Hat	Hut	3	1
79	Heart	Herz	4	1
80	Helmet	Helm	4	1
81	Horse	Pferd	5	1
82	House	Haus	4	1
83	Jar	Marmeladenglas	14	5
84	Ladder	Leiter	6	2
85	Lamp	Glühbirne	9	3

86	Leaf	Blatt	5	1
87	Lettuce	Salat	5	2
88	Lips	Mund	4	1
89	Lipstick	Lippenstift	11	3
90	Lizard	Eidechse	8	3
91	Lock	Schloss	7	1
92	Magnet	Magnet	6	2
93	Mailbox	Briefkasten	11	3
94	Man	Mann	4	1
95	Microphone	Mikrofon	8	3
96	Microscope	Mikroskop	9	3
97	Mouse	Maus	4	1
98	Mushroom	Pilz	4	1
99	Necklace	Kette	5	2
100	Needle	Nadel	5	2
101	Onion	Zwiebel	7	2
102	Orange	Orange	6	3
103	Paintbrush	Pinsel	6	2
104	Pan	Pfanne	6	2
105	Pants	Hose	4	2
106	Paperclip	Büroklammer	11	4
107	Pen	Kugelschreiber	14	4
108	Pencil	Bleistift	9	2
109	Pencilsharpener	Anspitzer	9	3
110	Phone	Telefon	7	3
111	Piano	Klavier	7	2
112	Pig	Schwein	7	1
113	Plate	Teller	6	2
114	Pot	Topf	4	1
115	Present	Geschenk	8	2
116	Radio	Radio	5	3
117	Rake	Haken	5	2
118	Robot	Roboter	7	3
119	Safe	Tresor	6	2
120	Safetypin	Sicherheitsnadel	16	5
121	Saw	Säge	4	2
122	Scissors	Schere	6	2
123	Screwdriver	Schraubenzieher	15	4
124	Shark	Hai	3	1

125	Shirt	Hemd	4	1
126	Shoe	Schuh	5	1
127	Shovel	Schaufel	8	2
128	Skateboard	Skateboard	10	4
129	Skis	Ski	3	1
130	Snake	Schlange	8	2
131	Sock	Socke	5	2
132	Spoon	Löffel	6	2
133	Stairs	Treppe	6	2
134	Statue	Statue	6	3
135	Stool	Hocker	6	2
136	Suitcase	Koffer	6	2
137	Sword	Schwert	7	1
138	Tape	Kassette	8	3
139	Teeth	Zahn	4	1
140	Tie	Schlips	7	1
141	Tomato	Tomate	6	3
142	Toothbrush	Zahnbürste	10	3
143	Trumpet	Trompete	8	3
144	Umbrella	Regenschirm	11	3
145	Vacuum	Staubsauger	11	3
146	Violin	Geige	5	2
147	Wheelbarrow	Schubkarre	10	3
148	Wheelchair	Rollstuhl	9	2
149	Woman	Frau	4	1
150	Yoyo	Jojo	4	2
